# Suppression of miR-330-3p alleviates DSS-induced ulcerative colitis and apoptosis by upregulating the endoplasmic reticulum stress components XBP1

**DOI:** 10.1186/s41065-020-00135-z

**Published:** 2020-05-09

**Authors:** Qifeng Chen, Xiaoming Fang, Ning Yao, Fang Wu, Biao Xu, Zhengguang Chen

**Affiliations:** 1 Department of Gastroenterology Surgery, Shulan(hangzhou) Hospital, No. 848, Road Dongxin, District Xiacheng, Hangzhou City, 310000 Zhejiang Province China; 2 Department of General Surgery, 903th hospital of PLA, Hangzhou City, 310000 Zhejiang Province China

**Keywords:** miR-330-3p, Ulcerative colitis, Apoptosis, Endoplasmic reticulum stress, XBP1

## Abstract

**Background:**

This study aimed to explore the biological activities of miR-330-3p in dextan sulphate sodium (DSS)-induced ulcerative colitis and apoptosis and the direct target of miR-330-3p in this process. HT-29 cells and male C57BL/6 mice were used to examine the function of miR-330-3p in vitro and in vivo, respectively. Expression of miRNA and mRNA was measured using quantitative real time PCR (qRT-PCR). Western blotting was used to measure the change of protein expression. Flow cytometry was used to determine cell apoptosis and luciferase assay was used to confirm the direct target of miR-330-3p.

**Results:**

miR-330-3p expression was increased by DSS in both HT-29 cells and mice. Upregulation miR-330-3p induced cell apoptosis, mice weight loss and ulcerative colitis in vivo, which could prevent by suppression of miR-330-3p. Cell apoptosis related protein expression, cleaved caspase-3 and cleaved PARP was also inhibited by miR-330-3p overexpression and elevated by miR-330-3p inhibition both in vitro and in vivo. Luciferase assay confirmed that 3′ untranslated region (3′-UTR) of XBP1 is the directed target of miR-330-3p and Western blotting results have showed that protein expression of XBP1 was decreased by miR-330-3p mimics and increased by miR-330-3p inhibitor.

**Conclusion:**

miR-330-3p is upregulated by DSS in both HT-29 cells and mice and promoted ulcerative colitis and cell apoptosis by targeting of 3′-UTR of XBP1, which is a key component of ER stress. Inhibition of miR-330-3p prevent DSS-induced ulcerative colitis and cell apoptosis mediated by upregulation of XBP1 expression.

## Background

Ulcerative colitis is a chronic, inflammatory disorder of the colonic mucosa which can generally extend to the entire colon in a continuous manner [1]. The underlying cause of this disease is still unknown. Many factors have been reported to influence ulcerative colitis, including genetic, environmental and immunological factors [2, 3]. Recent studies have revealed that cellular stress signaling including oxidative stress, mitochondrial hemostasis, autophagy and endoplasmic reticulum (ER) stress contributed to regulation of intestinal epithelial cell function [4].

ER plays a critical role in protein synthesis, folding and modification and calcium storage [5].Misfolded- or unfolded-protein is accumulated in the ER lumen when ER function is dysregulated, which is also called ER stress [6]. Inositol-requiring enzyme 1 (IRE1) is a conserved ER stress sensor and activation of IRE1α can promote mRNA translation to produce X-box–binding protein 1 (XBP1) [7]. XBP1 is a trancription factor, which is related to ER quality control and ER-associated proteolysis [8, 9]. Deletion of XBP1 in mice impaired antimicrobial function and worsened rectal bleeding induced by dextran sulphate sodium (DSS) [10]. When XBP1 was activated by HLJ2, an XBP1 agonist, decreased weight loss, disease activity index (DAI), colon contracture and reduced production of the inflammatory cytokines TNF-α, IL-1β, and IL-6 was observed in mouse model of DSS-induced colitis [11], demonstrating that XBP1 plays a protective role in DSS-induced ulcerative colitis.

microRNAs are a class of small, noncoding RNAs which contain ∼22 nucleotides in length and promote target mRNA cleavage or suppress protein expression by binding the 3′ untranslated region (3′-UTR) of target mRNA [12]. With the development of the in-silico techniques, it is found that single nucleotide polymorphisms is the main cause to induce alternation of miRNAs and their binding sites, resulting in the disease progression [13–15]. A number of studies have examined miRNA expression in intestinal tissues and several miRNAs have been identified to participate the pathological process of ulcerative colitis [16]. For example, Upregulation of miR-15 in ulcerative colitis could activate NF-κB signaling pathway through targeting adenosine A2 receptor, which worsened the ulcerative colitis [17]. Overexpression of miR-141 reduced MMP-2 and MMP-9 levels via direct downregulation of CXCL5 expression, indicating a protective effects on ulcerative colitis [18]. Our preliminary data showed that miR-330-3p expression was upregulated in DSS-induced ulcerative colitis, but the mechanism is still unclear. This study aims to explore the pathological function of miR-330-3p in DSS-induced ulcerative colitis and the underlying signaling pathway involved in this process, which will provide a new diagnostic biomarker and a potential therapeutic target for ulcerative colitis.

## Results

### miR-330-3p was upregulated in DSS-induced ulcerative colitis in mice

**Fig. 1:**
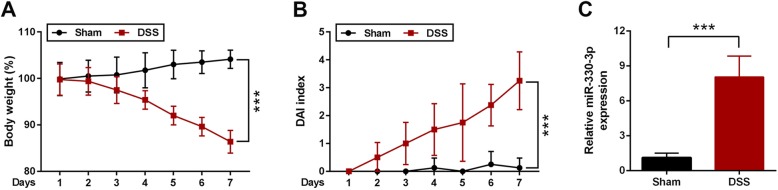
miR-330-3p expression was increase by DSS in mice. **a** Body weight was reduced in mice treated with DSS; **b** DAI score was elevated in mice treated with DSS; **c** miR-330-3p expression was upregulated in in mice treated with DSS. ** *p* < 0.01 versus (vs.) Sham; *** *p* < 0.005 vs. Sham

After treated with 4% of DSS for 7 days, the body weight of mice was greatly reduced in DSS treated mice compared with mice given normal drinking water (Fig. 1a). DAI score was higher in DSS treated mice than that in control group (Fig. 1b). miR-330-3p expression was dramatically increased in DSS treated mice compared with mice given normal drinking water (Fig. 1c).

### miR-330-3p induced cell apoptosis in DSS treated HT-29 cells

**Fig. 2:**
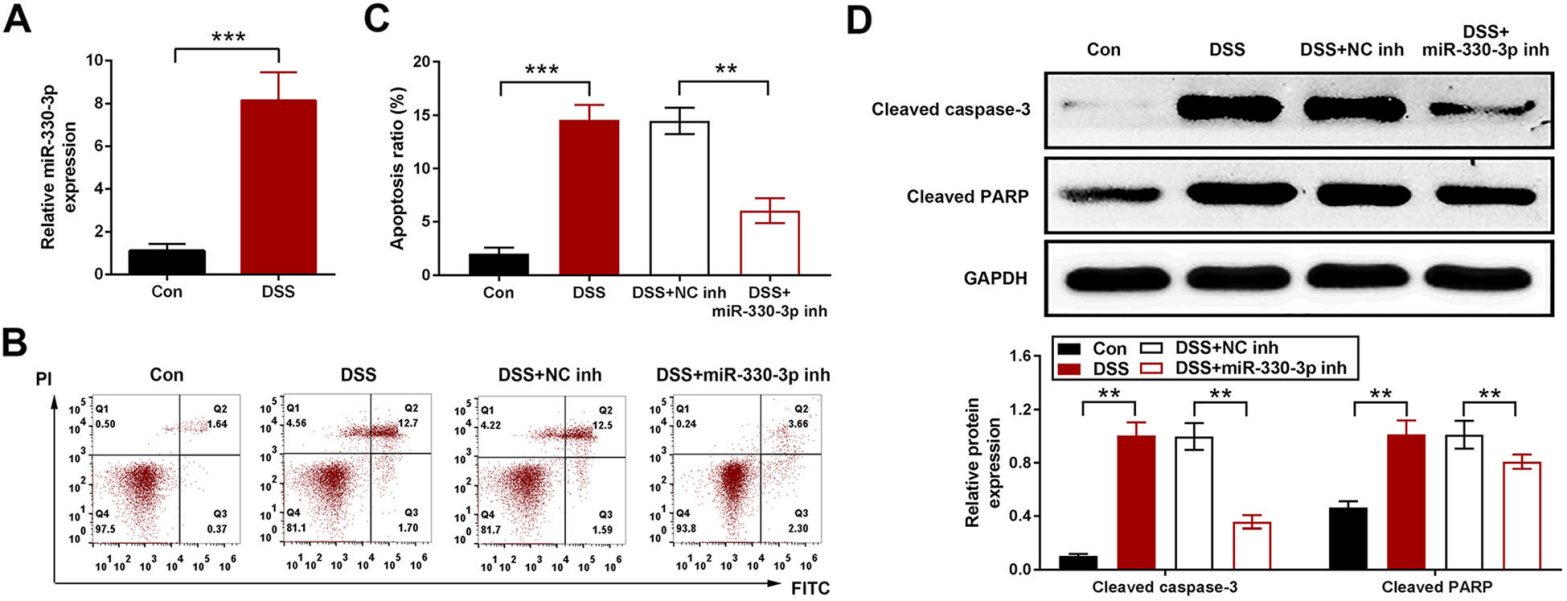
Upregulation of miR-330-3p induced HT-29 cell apoptosis. **a** miR-330-3p expression was increased by DSS in HT-29 cells; **b** miR-330-3p inhibitor inhibited cell apoptosis induced by DSS in HT-29 cells; Flow cytometry data shown as two parameter dot-plots; **c** miR-330-3p inhibitor inhibited cell apoptosis induced by DSS in HT-29 cells; statistically data shown as column; **d** Upregulation of of cleaved caspase-3 and cleaved PARP expression was inhibited by miR-330-3p inhibitor. ** *p* < 0.01 vs. Con. or DSS + NC inh; *** *p* < 0.005 vs. Con. or DSS + NC inh

In HT-29 cells, miR-330-3p was upregulated by 2% of DSS (Fig. 2a). Cell apoptosis was increased in DSS treated cells and this increase was inhibited in cell transfected with miR-330-3p inhibitor (Fig. 2b and c). The apoptosis related proteins, cleaved caspase-3 and cleaved PARP were determined by Western blotting. The expression of these two proteins was upregulated by DSS. In the cells transfected with miR-330-3p inhibitor, the expression of cleaved caspase-3 and cleaved PARP was reduced compared with the cells treated with DSS (Fig. 2d).

### XBP1 was the direct target of miR-330-3p

**Fig. 3:**
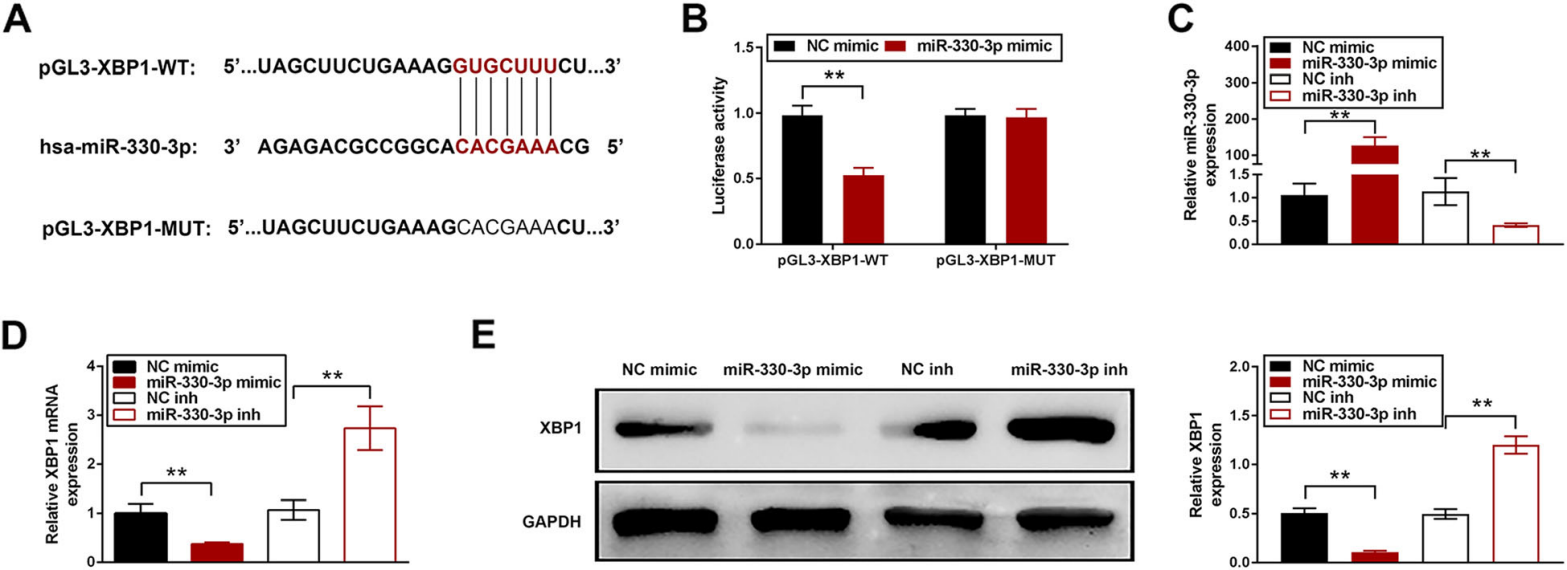
3′-UTR of XBP1 was the direct target of miR-330-3p. **a** The putative binding sites between miR-330-3p and 3′-UTR of XBP1; **b** Luciferase activity was decreased in co-transfection of pGL3-XBP1-WT and miR-330-3p mimic but no change in co-transfection of pGL3-XBP1-MUT and miR-330-3p mimic; **c** miR-330-3p expression was increased by miR-330-3p mimic and decreased by miR-330-3p inhibitor; **d** Protein expression was reduce by miR-330-3p mimic and induced by miR-330-3p inhibitor. ** *p* < 0.01 vs. NC mimic or NC inh; *** *p* < 0.005 vs. NC mimic or NC inh

TargetScan (http://www.targetscan.org/) was used to predict the potential target of miR-330-3p. The results demonstrated that there was a miR-330-3p binding site in the 3′-UTR of XBP1 (Fig. 3a). Co-transfection of XBP1 3′-UTR and miR-330-3p mimic showed a reduction of luciferase activity compared to negative group control while no significant difference was observed in cells transfected with XBP1 3′-UTR scramble sequence and miR-330-3p mimic (Fig. 3b). The expression of miR-330-3p was elevated by miR-330-3p mimic and repressed by miR-330-3p inhibitor (Fig. 3c). Protein expression of XBP1 was reduced by miR-330-3p mimic and induced by miR-330-3p inhibitor (Fig. 3d).

### Knockdown of miR-330-3p alleviated DSS-induced ulcerative colitis

**Fig. 4:**
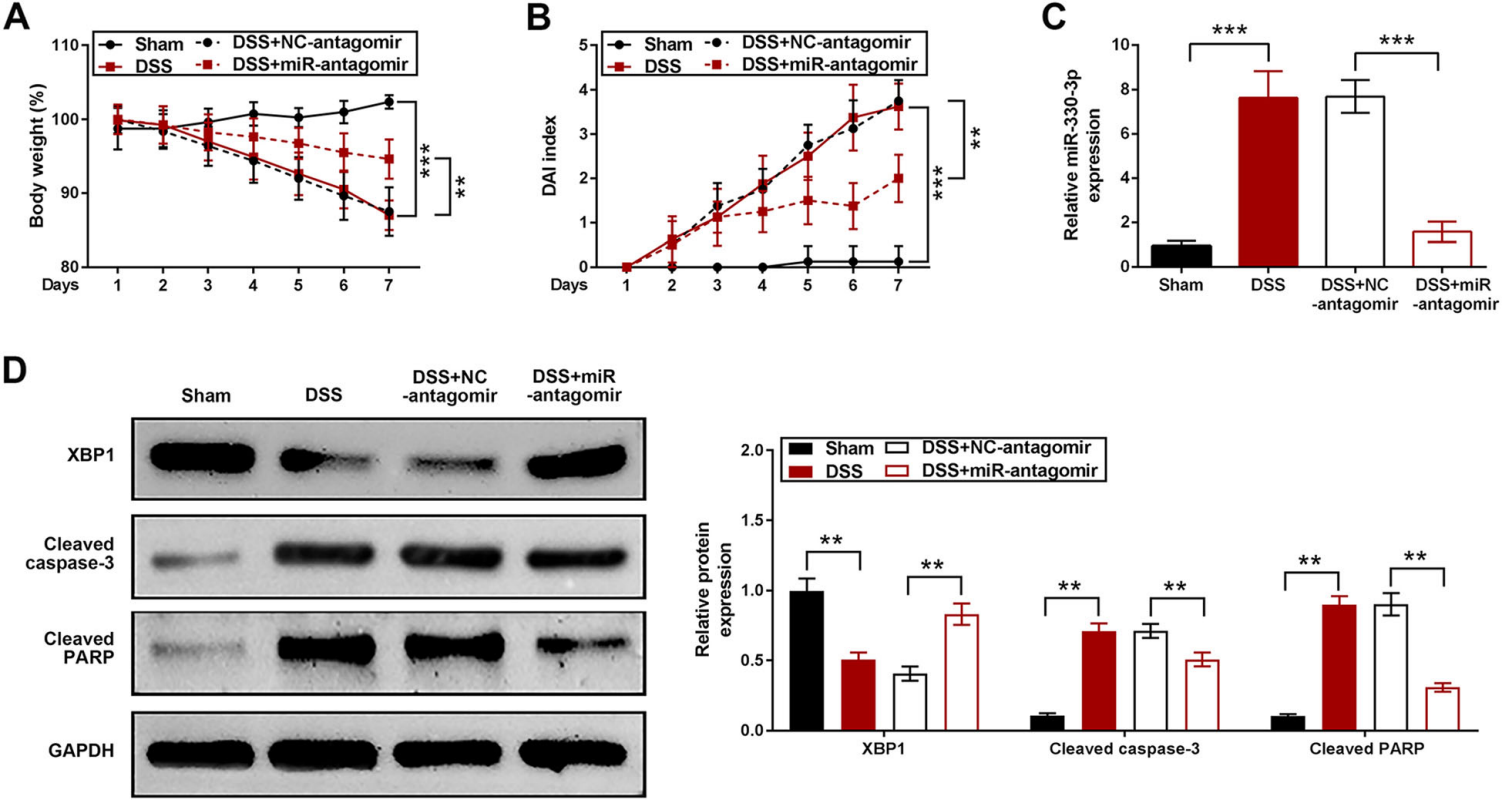
miR-330-3p antagomir alleviated DSS-induced ulcerative colitis in mice. **a** Body weight loss induced by DSS was prevented by miR-330-3p antagomir; **b** Elevation of DAI score induced by DSS was decreased by miR-330-3p antagomir; **c** Upregulation of miR-330-3p was induced by DSS was inhibited by miR-330-3p antagomir; **d** Change of XBP1, cleaved caspase-3 and cleaved PARP expression induced by DSS was downregulated by miR-330-3p antagomir; ** *p* < 0.01 vs. Sham or DSS + NC-antagomir; *** *p* < 0.005 vs. Sham or DSS + NC-antagomir

Knockdown of miR-330-3p was achieved by intraperitoneal injection of miR-330-3p antagomir. The body weight was reduced in mice treated with DSS, which was prevented by miR-330-3p antagomir (Fig. 4a). DAI score was increase in mice treated with DSS and this increase was smaller in mice pre-injected with miR-330-3p antagomir (Fig. 4b). Upregulation of miR-330-3p expression was observed in DSS treatment group and injection of miR-330-3p antagomir reduced the miR-330-3p expression compared with negative control group (Fig. 4c). XBP1 expression was reduced in mice treated with DSS (Fig. 4d). miR-330-3p antagomir showed a significant upregulation of XBP1 expression compared with negative control group (Fig. 4d). Both cleaved caspase-3 and cleaved PARP expression was upregulated by DSS in mice and DSS-induced overexpression of cleaved caspase-3 and cleaved PARP reduced by pre-injection of miR-330-3p antagomir (Fig. 4d).

## Discussion

Ulcerative colitis is a kind of inflammatory bowel disease and the common clinical symptoms are: diarrhea, rectal bleeding, abdominal pain and weight loss etc. [19]. Various chemicals have been introduced to induce colitis models in laboratory studies and the most widely used are trinitrobenzene sulfonic acid, oxazolone induced-colitis and DSS [20]. In this study, DSS was used to induce the ulcerative colitis and the results indicated that weight loss and DAI increase was observed, which are typical symptoms of ulcerative colitis. The number of cell apoptosis was also increased in HT-29 cells, an intestinal epithelial cell line. Therefore, this model can be used for further study.

Many studies have revealed that miRNAs were linked to the pathogenesis of colitis [21, 22]. For example, reduction of miR-124 in ulcerative colitis promoted intestinal inflammation mediated by transducer and activator of transcription 3 [23]. Overexpression of miR-31 and miR-155 regulated interleukin-13 signaling pathway via targeting interleukin-13 receptor α-1 in ulcerative colitis [24]. The previous study has found that miR-330-3p was overexpressed in ulcerative colitis [25]. However, no publication has reported the role of miR-330p-3p in ulcerative colitis. Data of the present study revealed that inhibition of miR-330-3p decreased HT-29 cell apoptosis and prevented weight loss and DAI elevation in mouse model, suggesting that suppression of miR-330-3p could ease the symptoms caused by ulcerative colitis, protecting the injury of ulcerative colitis.

As mentioned, the biological function of miRNAs was achieved through bind to 3′-UTR of target mRNA. The 7 nucleotides “seed region”, 2 to 8 at the 5′ end, of miRNA is the key point to target recognition [26, 27]. Target prediction results from TargetScan demonstrated that 3′-UTR of XBP1 bound at 3 to 9 at 5′ end of miR-330-3p and a perfect Watson-Crick match [28] was observed between miRNA and mRNA at the binding site, indicating that 3′-UTR of XBP1 is probably the direct target of miR-330-3p. To confirm this prediction, luciferase assay was conducted [29]. The results of luciferase assay and Western blotting manifested that overexpression of miR-330 reduced XBP1 level. These data validated that miR-330-3p is able to target 3′-UTR of XBP1.

XBP1 is an important transcription factor, which is required for ER stress [30]. Published data have manifested that activation of XBP1 may produce an anti-inflammatory effect [31, 32]. In this study, XBP1 expression was reduced via upregulation of miR-330-3p. Inhibition of miR-330-3p showed a significant upregulation of XBP1 expression and reduction of weight loss and DAI elevation in DSS-induced colitis, which is consistent with the previous finding that activation of XBP1 inhibited the pathogenesis of ulcerative colitis [11]. Therefore, XBP1 could be a therapeutic target for ulcerative colitis.

## Conclusion

In conclusion, miR-330-3p was upregulated by DSS in both HT-29 cells and mice and promoted ulcerative colitis and cell apoptosis by targeting of 3′-UTR of XBP1, which is a key component of ER stress. Inhibition of miR-330-3p prevent DSS-induced ulcerative colitis and cell apoptosis and increased XBP1 expression, suggesting that miR-330-3p might be a diagnostic biomarker of ulcerative colitis, and that XBP1 could be a potential therapeutic target for ulcerative colitis. However, all data in the present study are preclinical results, so further clinical study are warranted to figure out the effects of miR-330-3p/XBP1 network in ulcerative colitis.

## Methods

### Cell culture and miRNA transfection

Human intestinal epithelial cell line, HT-29 cells were purchased from the American Type Culture Collection (ATCC, USA). The cells were cultured with DMEM which was supplemented with heat inactivated fetal bovine serum (FBS; 10%, v/v; Gibco, USA), penicillin (100 U/ml, Gibco, USA), streptomycin (100 μg/ml, Gibco, USA) and incubated under the environment of 5% CO_2_ at 37 °C. DSS (Sigma-Aldrich, USA) was dissolved in sterile water. HT-29 cells were seeded in sterile 6-well plates. When cell confluence reached to about 80%, cells were treated with 2% of DSS or sterile water for 24 hours (h) [10]. Each experiment was performed in triplicate.

miR-330-3p mimic or its negative control (NC mimic), miR-330-3p inhibitor (inh) or its negative control (NC inh) (Ribobio, China) was dissolved in sterile phosphate-buffered saline (PBS; Jijinchem, China). 70 μl of this solution was gently mixed with 30 μl of Lipofectamine™ 2000 (Invitrogen, USA) and incubated at room temperature for 15–20 minutes (min) to form the complex according to the manufacturer’s instructions. HT-29 cells were seeded in sterile 6-well plates. When cell confluence reached to about 60%, 100 nM of the complex was transfected into cells. After transfection for 24 h, DSS were added and incubated for 24 h as described above. Each experiment was performed in triplicate.

### Animal model

C57BL/6 mice (male, 18–20 g, 6–8 weeks) were purchased from Guangdong Medical Laboratory Animal Center and kept under the environment of 23 ± 2 °C, 55 ± 15% humidity, 12 h light/12 h dark cycle. miR-330-3p antagomir or its negative control (NC) was dissolved in sterile PBS and mixed with Lipofectamine™ 2000 (Invitrogen, USA) to form the complex (1 nmol/200 μl of antagomir) as described above. Mice were assigned into 4 groups (*n* = 8 mice/group) randomly: Group I (Sham): mice were given normal drinking water; Group II (DSS): mice administered with drinking water containing 4% of DSS for 1 week; Group III (DSS + NC-antagomir): mice were administered with 200 μl of NC antagomir mixture by tail vein injection twice per week for 1 week and then administered with drinking water containing 4% of DSS for 1 week; Group IV (DSS + miR-antagomir): mice were administered with 200 μl of miR-330-3p antagomir mixture by intraperitoneal injection twice per week for 1 week and then administered with drinking water containing 4% of DSS for 1 week. DAI was calculated as follows: DAI = (weight loss score + stool characters score + bleeding score)/3 [33]. The mice weights were calculated before killed by overdose of pentobaribital sodium (100 mg/kg) and the colon tissues were collected for further analysis.

All experiment procedures were carried out following the ethical standards under a protocol approved by the Ethics Committee of Shulan(hangzhou) Hospital, and were executed conforming to the Guide for the Care and Use of Laboratory Animals published by the US National Institutes of Health (No. 85–23, 1996) [34].

### RNA extract and quantitative real time PCR (qRT-PCR) assay

Trizol reagent (Invitrogen, USA) was used to extract total RNA. Reverse transcription was achieved using the PrimeScript RT Master Mix (TaKaRa, Dalian, China) according to the manufacturer’s instruction. The relative RNA expression was examined by qRT-PCR assay using the SYBR Premix Ex Taq II Kit (TaKaRa) on the StepOnePlus system (Applied Biosystems, CA, USA). The data were calculated by means of the 2^−ΔΔCt^ method. The primer sequences (Invitrogen, USA) used in this study were shown as Table 1.

**Table 1:** Primer sequences for RT-qPCR

Gene	Primer sequences
U6	Forward: CGCTTCGGCAGCACATATAC
	Reverse: TTCACGAATTTGCGTGTCAT
miR-330-3p	Forward: CAACTGCCTCTCTGGGCCTG
	Reverse: CTGCAGAGAGGCAGCGCTG
XBP1	Forward: ACATCTTCCCATGGACTCTG
	Reverse: TAGGTCCTTCTGGGTAGACC
GAPDH	Forward: CCACATCGCTCAGACACCAT
	Reverse: CCAGGCGCCCAATACG

### Western blotting

Protein lysates were extracted using RIPA cell lysis buffer (Beyotime, China) and protein concentration was measured using BCA protein assay kit (Beyotime, China). Total protein (50 μg) were electrophoresed on 10% polyacrylamide gel (SDS-PAGE) and transferred to PVDF membranes (Millipore, USA) followed by blockade with 5% of milk for 1 h. Membrans were sequentially probed with the indicated primary antibodies: Cleaved caspase-3 (CST9664, 1:1000 dilution), Cleaved PARP (CST5625, 1:1500 dilution), XBP1 (CST40435, 1:500 dilution) and GAPDH (CST2118, 1:5000 dilution). After incubated with primary antibodies overnight at 4 °C, the membranes were incubated with the appropriate secondary antibodies (Cell Signaling Technology, USA). Finally, the bands were detected using SignalFire™ ECL Reagent (Cell Signaling Technology, USA).

### Plasimid and luciferase reporter assay

This protocol was followed the published paper [35]. Briefly, 3′-UTR of XBP1 containing wide type (WT) and scrambled (MUT) miR-330-3p binding sequence were inserted downstream of the firefly luciferase gene in pGL3 to generate the pGL3-XBP1-WT or pGL3-XBP1-MUT plasmid respectively. The constructed plasmids were co-transfected into HT-29 cells with NC mimic or miR-330-3p mimic using Lipofectamine 2000. After 24 h, luciferase activity was assayed using the Luciferase Reporter Assay kit (Abcam, UK) according to the manufacturer’s protocol.

### Cell apoptosis by flow cytometry

Flow cytometry was used to detect HT-29 cell apoptosis using an annexin V-FITC apoptosis detection kit (Beyotime Biotechnology) according to the manufacturer’s protocol. The stained samples were analyzed with FACSCalibur (BD, New Jersey, USA). The data of apoptotic cells were shown as two parameter dot-plots.

### Statistical analysis

SPSS 10.0 was used to perform the statistical analysis in this study. Student’s *t*-test was used to compare the difference between two groups. Comparisons of more than two groups was performed using one-factor analysis of variance. All data were demonstrated as means ± standard error of means (S.E.M.). *p* < 0.05 represents statistically significant.

## Data Availability

All data generated or analyzed during this study are included in this published article.

## Abbreviations

qRT-PCR: Quantitative real time PCR
DSS: Dextran sulphate sodium
ER: Endoplasmic reticulum
IRE1: Inositol-requiring enzyme 1
XBP1: X-box–binding protein 1
DAI: Disease activity index
3′-UTR: 3′ untranslated region
